# A Social Media Outage Was Associated with a Surge in Nomophobia, and the Magnitude of Change in Nomophobia during the Outage Was Associated with Baseline Insomnia

**DOI:** 10.3390/clockssleep4040040

**Published:** 2022-09-27

**Authors:** Haitham Jahrami, Feten Fekih-Romdhane, Zahra Saif, Nicola Luigi Bragazzi, Seithikurippu R. Pandi-Perumal, Ahmed S. BaHammam, Michael V. Vitiello

**Affiliations:** 1 Ministry of Health, Manama 410, Bahrain; 2 Department of Psychiatry, College of Medicine and Medical Sciences, Arabian Gulf University, Manama 323, Bahrain; 3 Department of Psychiatry “Ibn Omrane”, Razi Hospital, Manouba 2010, Tunisia; 4 Faculty of Medicine of Tunis, Tunis El Manar University, Tunis 1068, Tunisia; 5 Industrial and Applied Mathematics (LIAM), Department of Mathematics and Statistics, York University, Toronto, ON M3J 1P3, Canada; 6 Saveetha Medical College and Hospitals, Saveetha Institute of Medical and Technical Sciences, Saveetha University, Chennai 602117, India; 7 Department of Medicine, College of Medicine, University Sleep Disorders Center, King Saud University, P.O. Box 225503, Riyadh 11324, Saudi Arabia; 8 The Strategic Technologies Program of the National Plan for Sciences and Technology and Innovation in the Kingdom of Saudi Arabia, P.O. Box 2454, Riyadh 11324, Saudi Arabia; 9 Psychiatry & Behavioral Sciences, Gerontology & Geriatric Medicine, and Biobehavioral Nursing, University of Washington, Seattle, WA 98195-6560, USA

**Keywords:** anxiety, insomnia, nomophobia, sleep problems, social media outage

## Abstract

We examined the immediate impact of a social media outage on nomophobia and associated symptoms using a longitudinal cohort design. Data were collected at two timepoints, baseline (T1) and during the social media outage of 4 October 2021 (T2). T1 was collected in August–September 2021 as part of the baseline of an ongoing study. The nomophobia questionnaire (NMP-Q), Generalized Anxiety Disorder-7 scale (GAD-7), and Athens insomnia scale (AIS) were administered to 2706 healthy participants from the general Bahraini population (56% females, mean age 33.57 ± 11.65 years). Approximately one month later, during the social media outage, 306 of the study participants were opportunistically assessed using the NMP-Q. At baseline, we found that nomophobia levels strongly correlated positively with both insomnia (*p* = 0.001) and anxiety symptoms (*p* = 0.001). This is the first report to examine the impact of a social media outage on nomophobia. Our findings indicate that symptoms of nomophobia increased significantly during a social media outage. Baseline insomnia scores predicted a surge in the global scores of nomophobia symptoms during a social media outage.

## 1. Introduction

One of the greatest innovations in information and communication technologies was the introduction of the mobile phone (MP) [1]. MPs were initially introduced as portable phones that could be used outside of one’s premises. However, rapidly, MPs were replaced by smart mobile phones (SMPs), which combined the functions of MPs and computers into one device [2]. SMPs offer more robust hardware capabilities and extensive mobile operating systems, which facilitate broad software, internet (including web browsing and social media access), and multimedia functions (including video, cameras, and games), besides core phone functions such as voice and text messages [3]. As a result of these increased benefits, SMP use and internet access continue to grow worldwide, and social media use is on the rise. In 2020, internet users worldwide used social media for an average of 145 min (2.5 h) per day [4]. Globally, there were an estimated four billion active mobile social media users as of October 2020 [4], over half of the world’s population. As an alternative to short message service (SMS)-based text messaging, social media apps have proven themselves to be advanced [4]. WhatsApp, which had two billion monthly users as of January 2021, is the most popular mobile messaging app. Facebook Messenger, ranked second, had 1.3 billion monthly active users last year [4].

This increased use of SMPs has not been without its adverse effects on mental health, particularly among individuals who rely on SMPs for all aspects of their daily life. One of these adverse effects is ‘nomophobia’ [5]. The term nomophobia is derived from the term ‘no mobile phone phobia’, and initially emerging as a reference to SMP dependence [6,7,8]. Nomophobia is defined as a psychological issue or anxiety related to not having access to an SMP [5]. King and his colleagues first described nomophobia concerning both computer use and SMP use; later, they concentrated on SMP use [9]. Nomophobia can be viewed as a situational phobia, similar to aerophobia (fear of flying), caused and activated by stimuli that provoke anxiety [10,11]. Because of being without or unable to use their SMP, individuals who have nomophobia may experience higher levels of anxiety, fear, and other symptoms [9].

Nomophobia and the availability of social media platforms are logically linked to each other [12,13]. However, evidence supporting this logical linkage is lacking. For instance, it remains unknown whether a social media outage (defined as a partial or complete shutdown of online services) worsens nomophobia. On 4 October 2021, social media networks including Facebook, WhatsApp, and Instagram experienced the largest and longest outage since 2008 [14,15]. The outage lasted from ~16:00 GMT until ~22:00 GMT, and was an excellent and rare opportunity to examine the impact of such outages on nomophobia symptomology [15].

Furthermore, several recent literature reviews have presented evidence that nomophobia negatively impacts mental health, increasing anxiety [16] and lowering sleep quality [17]. These reviews have also explored the links between nomophobia and anxiety and sleep quality and symptoms of insomnia. The effect on anxiety may well follow the typical phobia–anxiety relationship, where the presence of the phobic object, or in this case, the absence of SMP functionality, results in increased anxiety [1,13,18]. Nomophobia’s impact on sleep quality and insomnia symptoms might be the direct result of the phobia, the associated anxiety, or other causes such as excessive night-time SMP use or exposure to blue light or electromagnetic radiation [1].

To the best of our knowledge, there are to date no studies regarding the effects of imposed inability to access social media platforms on nomophobia and its relationship with anxiety and insomnia. The 4 October 2021 social media outage provided us with an opportunity for such an examination. The present study aimed to examine, in a longitudinal cohort design, the immediate impact of the great social media outage of 4 October 2021 on nomophobia and its association with baseline anxiety and insomnia levels.

## 2. Results

At baseline, the mean age, BMI, and SMP use per day of the study participants were as follows: 33.57 ± 11.65 years, 24.96 ± 4.61 kg/m^2^, and 6.10 ± 2.90 h/d., respectively. Additionally, at baseline, the mean NMP-Q was 77.38 ± 26.38, the mean GAD-7 was 8.44 ± 4.96, and the mean AIS was 6.89 + 4.5. Approximately 22% of the participants had a severe level of nomophobia, about 12% had GAD, and 56% had insomnia. Samples at T1 and T2 were similar for all personal characteristic variables of interest; detailed results are presented in Table 1.

The NMP-Q and its dimensions are broken down by time points in Table 2. Paired samples *t*-test showed that total NMP-Q was significantly higher at T2 (*p* = 0.001, Cohen’s d = 0.57). In terms of the dimensions of nomophobia, all four dimensions showed significant elevation at T2.

Table 3 provides the results of the correlation analysis between study variables. The strongest correlation was found between NMP-Q (T1) and NMP-Q (T2) (r = 0.53; *p* = 0.001). Weaker correlations were observed between AIS and GAD-7 (r = 0.25; *p* = 0.001) and between NMP-Q (T1) and GAD-7 (r = 0.14; *p* = 0.001).

AIS at baseline (T1) successfully predicted the change in nomophobia symptoms defined as NMP-Q symptoms during social media outage (T2–T1). However, GAD at baseline (T1) did not predict the change in nomophobia symptoms defined as NMP-Q symptoms during the social media outage (T2–T1). Detailed results are presented in Table 4.

AIS at baseline (T1) successfully predicted the change in two (out of four) dimensions of nomophobia symptoms: NMP-Q Dimension 3: (D3) Not being able to access information and NMP-Q Dimension 4: (D4) Giving up convenience, with *p*-values of 0.03 and 0.006, respectively. However, GAD at baseline (T1) did not predict the change in any of the dimensions related to nomophobia symptoms defined as NMP-Q symptoms during the social media outage (T2–T1). Detailed results are presented in Table 5. 

## 3. Discussion

The current study offers a rare and uniquely measured insight into the impact of social media’s presence/absence on the lives of SMP users. The main finding was that a sudden loss of access to social media platforms caused an immediate surge in nomophobia. At baseline, our results showed that nomophobia was associated with increased SMP use duration, anxiety symptoms, and insomnia. It is very important to recognize that the survey period overlaps with the COVID-19 pandemic, which changed both mobile phone use and sleep issues. A recent review estimated that, during the COVID-19 pandemic, the global prevalence of sleep issues was 40% (38; 44%) [19]. Similarly, it is important to concede that screen time has significantly increased during the COVID-19 pandemic [20,21], which is acknowledged when interpreting the results.

As expected, the social media outage resulted in a significant increase in nomophobia symptoms, with a particular enhancement in fear of losing the connectedness that SMPs allow and not being able to access information. This is an interesting and unique finding, showing for the first time that a social media outage is associated with a worsening of nomophobia symptoms, contrary to most previous studies that did not differentiate the types of online activities and applications when dealing with the detrimental effects of the internet and SMP addiction [22,23]. A person with high social anxiety transfers most of their social activities, including developing strong friendships, to the virtual world, where they feel safer and more comfortable than in reality [24]. Nomophobia appears to be associated with insomnia, loneliness, social phobia, social anxiety, and social media addiction [25,26,27,28]. Moreover, our study findings revealed a significant association of nomophobia with time spent per day using a smartphone. Previous research showed that students who spent 5–10 h per day on a smartphone had significantly higher levels of nomophobia (97.33 ± 20.03) compared with the students who spent 1–5 h per day (83.55 ± 22.79) [29]. Pavithra and colleagues also found a significant association of nomophobia with the duration of SMP usage [30]. Social media use is high among young people with social dependency, especially those who use smartphones. Those with high social media dependence levels also have high addiction rates, according to Salehan and Negahban [31].

At baseline, we found that nomophobia significantly correlated with both generalized anxiety and insomnia symptoms, in line with previous studies [26,32,33]. A Malaysian study of addiction to SMPs among undergraduates reported that usage of SMPs was positively correlated with anxiety [34]. Earlier research suggested that there may be a relationship between addiction to social media and insomnia [9,35]. Several previous studies found a positive association between nomophobia and insomnia [5,13,18,36,37].

In two meta-analyses of insomnia sex differences, it was found that females who were 15–30 years old were approximately 1.2 times more likely to have insomnia than males, while in middle age (31–64 years), they were 1.4 times more likely to have insomnia, suggesting that females have a slightly increased rate of insomnia [38,39]. Nonetheless, nomophobia was not predicted by age or sex in meta-regressions models [5]. Thus, combining both findings, a consideration of both nomophobia and insomnia symptoms is needed for both women and men equally.

During normal times (no outage), social media use is typically an ongoing process; there is no clear beginning or end to the use of social media. Some individuals have difficulty resisting responding in a way that could interfere with normal daily functioning. Interactions on social media may also include a “waiting” element (e.g., sending out a message and waiting for a response) [40]. When a person is preoccupied and waiting for replies from others, the routine may be disrupted. This is particularly important for sleep routine; if an individual waits for social interaction during normal sleep hours, their bedtime may be delayed, causing their sleep rhythm to be out of synchrony [13]. A desynchronized sleep cycle can affect sleep quality and cause insomnia [34]. In addition, light-emitting screens in smartphones may suppress the sleep-promoting hormone melatonin, which is normally elevated before bedtime, and disrupt sleep patterns [34,41]. AIS at baseline (T1) successfully predicted the change in nomophobia symptoms defined as NMP-Q symptoms during the social media outage (T2–T1). Recent research showed that nomophobia intensity can be successfully assessed by sleep dissatisfaction [28]. Finally, social media use may result in psychological stimulation and anxiety; for example, a person’s mood may become elevated and excited by social media interactions, and these emotional states may interfere with sleep [18,41,42]. It has been shown that developing good sleep hygiene leads to better sleep quality, which suggests further consideration of specific aspects of internet use on sleep health [34]. Additionally, healthcare providers should consider implementing effective programs promoting sleep hygiene information in the context of nomophobia and social media addiction [43]. Special attention needs to be given to special population groups, e.g., those who have psychiatric disorders, especially existing anxiety disorders, and those individuals who engage in sedentary lifestyles. Previous research showed that obese participants had a higher rate of poor sleep, which was closely related to mood disturbance and a poor social life [44].

The primary strength of the current research is that the nature of the longitudinal design and large sample size allowed for comparisons over time for the same person or group of people. Longitudinal studies are often obstructed by loss to follow-up, which may lead to bias in the results. In this particular study, as follow-up was restricted to a very narrow window of recruitment, i.e., during the few hours of social media outage, the response rate was reasonable. It is unprecedented to collect data about nomophobia during a social media outage and provide novel findings on a relatively new and under-explored notion. The greatest limitations of the present research are the relatively small portion of the T1 sample that provided data at T2 and that only nomophobia was measured at T2. Additional limitation is that this study was conducted during the COVID-19 pandemic, which may have influenced the results because of the constant need to be updated with governmental announcements and status updates of the pandemic. The generalizability (external validity) of research findings refers to the extent to which they can be applied in different settings than those in which they were initially tested. Thus, more research in the field is needed to better understand the long-term effects of social media outages (or indeed, internet outages) on nomophobia and related symptoms. Another limitation of the present analysis is the lack of a clear analysis of the professional profile of the participants. It would be logical to assume that people whose jobs (e.g., public relations officers) need constant mobile use could experience greater nomophobia in the event of an outage. Thus, we encourage future studies to focus on the professional profile because it is important to differentiate between a surge in nomophobia symptoms and a legitimate interference with professional activity.

## 4. Materials and Methods

### 4.1. Study Design and Setting

Crowdsourcing platforms like Amazon Mechanical Turk are not traditionally used for research in some countries. Although crowdsourcing involves soliciting volunteers through an open call, the process is now being called ‘social media crowdsourcing’ [45] owing to the widespread use of social media platforms to solicit volunteers. Ninety-six studies in a systematic review and meta-analysis showed that crowdsourcing approaches can be a viable and effective means of improving knowledge [46]. Using social media and mobile phone short messaging services, we mimicked a crowdsourcing platform by soliciting responses to self-administered, structured questionnaires from a large pool of participants. A longitudinal study design was used to collect data for the present study. The present report is part of a large ongoing study that examines the longitudinal association between nomophobia and psychological disorder. Data for the current research were collected at two different time points: data were collected from each participant at baseline (T1 = during normal social media use, i.e., August–September 2021) and again from a subset of the T1 participants during a major social media network outage (T2 = Monday, 4 October 2021, between 20:00 and 22:00 GMT).

All data were collected using web-based, self-administered, structured questionnaires distributed via instant messaging groups and social media ads by key contact persons. We encouraged survey participants to forward the link to friends and relatives who might be eligible. To improve the standard of research design and documentation, the Strengthening the Reporting of Observational Studies of Epidemiology (STROBE) recommendations were implemented in this study [47]. The electronic survey was created using the Checklist for Reporting Results of Internet E-Surveys [48]. We used mandatory fields for all variables in our survey to avoid missing data. To allow comparison between datapoints, participants were instructed to generate a secure, anonymous, universally unique identifier (UUID) 128-bit label [49] using an online free tool during datapoint T1 and to reuse it throughout the study at subsequent time points. The UUID was used to match longitudinal responses of the participants from various timepoints.

### 4.2. Participants and Sample Size

Healthy participants from the general Bahraini population were selected from a convenience, non-probability sample of individuals of both sexes, aged ≥18 years, who owned at least one mobile device, and were willing to participate in the study. We excluded participants with a formal diagnosis of any neuropsychiatric disorder using a direct questioning skip logic protocol.

**Figure 1:**
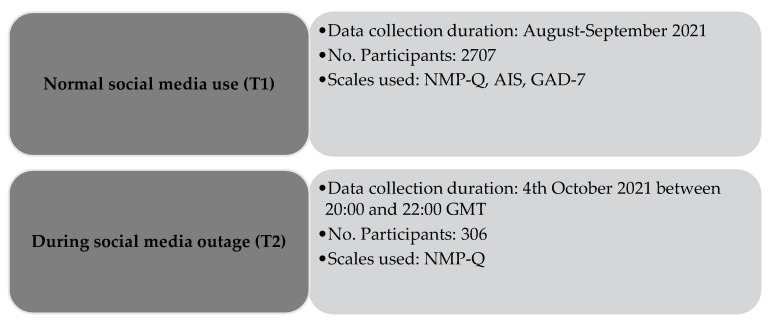
Flow diagram of participants’ recruitment. Notes: NMP-Q = Nomophobia Questionnaire (NMP-Q); AIS = Athens Insomnia Scale, GAD-7 = General Anxiety Disorder-7.

A total of 2706 participants (56% females) were included at the start of the study (T1). At T2, an opportunistic effort to assess the sample was made via active platforms and personal contact. This resulted in 306 (11%) participants of the initial sample providing usable responses. Sample characteristics of the T2 sub-sample were similar to the T1 sample. Figure 1 demonstrates a flow diagram of participants’ recruitment.

### 4.3. Ethical Considerations

The Helsinki Declaration of 1964 and its modifications directed all processes of the current research. This study was evaluated and authorized by the Research Ethics Committee of the Ministry of Health in Bahrain (REC/21/5363). The anonymity of the data was assured, and participants were informed that their provided information would be used only for research purposes. They were informed about the study’s aims and electronic written informed consent was obtained from each participant. Participants were informed that their participation in this survey is voluntary and that they may refuse to take part in the research or exit the survey at any time. No monetary or non-monetary incentives were offered in reward for participation. There was no specific grant from any funding agency in the public, commercial, or not-for-profit sectors to support this research.

### 4.4. Data Sources and Measurement

The data were collected using a structured survey. Pre-survey screening questions, sociodemographic questions, questions about personal SMP, the Nomophobia Questionnaire (NMP-Q) [11], the generalized anxiety disorder symptoms assessment (GAD-7) [50], and the Athens Insomnia Scale [51] were all included in the electronic survey.

#### 4.4.1. Measures of Sociodemographic and Anthropometry

We asked about age, sex, weight, height, and self-reported health information in the demographic question set. Based on a person’s weight and height, BMI was calculated by dividing the body mass by the square of the height [52]. BMI is expressed in kilograms per square meter (kg/m^2^), with body mass in kilograms and height in meters. Underweight was defined as ≤18.5 kg/m^2^, normal weight as 18.5–24.9 kg/m^2^, overweight as 25–29.9 kg/m^2^, and obese as ≥30 kg/m^2^ [52].

#### 4.4.2. Nomophobia, Anxiety, and Insomnia Measures

Nomophobia is defined as the anxiety or phobia of not having a smartphone or handheld mobile device. Using the NMP-Q, the severity of nomophobia was assessed. The NMP-Q includes 20 questions, each of which is scored on a seven-point Likert-like scale ranging from 1 (“strongly disagree”) to 7 (“strongly agree”) [11]. NMP-Q has four dimensions: (1) not being able to communicate, (2) losing connectivity, (3) not having access to information, and (4) giving up convenience [11]. There are four categories of overall scores: <20 no nomophobia; 21–59 mild nomophobia; 60–99 moderate nomophobia; and 100–140 severe nomophobia [11]. Our study used an Arabic validated version of the NMP-Q, which has excellent psychometric properties, with a Cronbach’s alpha coefficient of 0.90 [53].

The Generalized Anxiety Disorder-7 (GAD-7) [50] was used to measure the severity of generalized anxiety symptoms among participants. The GAD-7 originally had a cutoff score of 10 (sensitivity of 89% and specificity of 82% for identifying GAD in the general population) [50]. Anxiety levels are interpreted as follows: 0–4: minimal anxiety, 5–9: mild anxiety, 10–14: moderate anxiety, and 15–21: severe anxiety [50]. Our study used an Arabic validated version of the GAD-7, which has good psychometric properties, with a Cronbach’s alpha coefficient of 0.83 [54].

The Athens Insomnia Scale (AIS) [51] was used to examine participants’ insomnia symptoms. This instrument uses diagnostic criteria established by the International Classification of Diseases (ICD-10) [55]. Eight items in the questionnaire measure sleep onset, early morning awakenings, sleep time, sleep quality, complaints, the stress associated with insomnia, and interference with daily activities [51]. Researchers validated the instrument on patients with insomnia and control participants aged 18–79 years. For insomnia to be diagnosed, a score of ≥6 on the AIS is required [51]. Our study used an Arabic validated version of the AIS, which has good psychometric properties, with a Cronbach’s alpha coefficient of 0.83 [56].

At T1, we obtained results for all three scales (i.e., NMP-Q, GAD-7, and AIS), while for T2, we obtained results for NMP-Q only. GAD-7 and AIS were not included in T2 for two main reasons. First, to encourage cooperation and response rate by only focusing on the most significant research scale (i.e., NMP-Q items). Secondly, because we needed to collect data during the outage event hours, a ‘now and here’ assessment of insomnia symptoms was simply not possible because the outage ended on the same day.

### 4.5. Statistical Analysis

We used descriptive statistics to report the demographic characteristics, NMP-Q, GAD-7, and AIS scores of the subjects. Continuous variables were reported as the arithmetic mean and standard deviation, while categorical variables were reported as frequency counts and percentages. To ensure that data from T1 and T2 are from similar procedures, independent samples *t*-test/or chi-square (χ^2^) was performed.

The difference between the study variables T1 and T2 was compared using the paired samples *t*-test. Using Cohen’s d, the effect size was estimated as follows: small = 0.20, moderate = 0.50, and large ≥ 0.80 [57].

The Pearson’s product–moment correlation coefficient was used to determine how strongly three variables (NMP-Q, GAD-7, and AIS) correlate linearly. Linear regression analysis was used for AIS (T1) and GAD (T1) to predict the change in NMP-Q score, defined as T2–T1. Linear regression analysis was also used for AIS (T1) and GAD (T1) to predict change in the four individual dimensions of NMP-Q score, defined as T2–T1.

Two-tailed tests were considered significant if the *p*-value was less than 0.05. The data were analyzed using the R statistical computing package 4.1.2 (Bird Hippie) [58]. Package ‘gbm’ was used to perform generalized boosted regression models in machine learning [59].

## 5. Conclusions

The digital age has brought with it a condition known as nomophobia, which arises from a pathological fear of being without access to SMP technology and connectivity. This is the first report to examine the impact of a social media outage on nomophobia. Our findings show that symptoms of nomophobia increased significantly during the outage, particularly concerns about access and connectivity. Nomophobia was closely associated with anxiety and insomnia symptoms at baseline. A social media outage is associated with a surge in nomophobia, and the magnitude of the change in nomophobia symptoms during the outage was associated with baseline insomnia scores.

## Figures and Tables

**Table 1:** Descriptive statistics of study participants.

Variable	T1 (N = 2706)	T2 (N = 306)	*p* *
Continuous data			
Age, years	33.57 ± 11.65	33.31 ± 11.36	0.7
BMI, Kg/m^2^	24.96 ± 4.61	24.65 ± 4.49	0.3
MPU, h/d	6.10 ± 2.90	6.14 ± 3.00	0.8
Categorical data			
Sex			
Female	1526 (56.39%)	167 (54.57%)	0.8
Male	1180 (43.61%)	139 (45.43%)	
Marital Status			
Married/In relationship	951 (35.14%)	114 (37.26%)	0.8
Single	1755 (64.86%)	192 (62.74%)	
Job Status			
Employed/Student	2342 (86.55%)	159 (86.27%)	0.9
Not Employed	364 (13.45%)	42 (13.72%)	
BMI Category			
Underweight	125 (4.62%)	19 (6.20%)	0.7
Normal weight	1409 (52.07%)	167 (54.57%)	
Over weight	854 (31.56%)	82 (26.79%)	
Obese	318 (11.75%)	38 (12.41%)	
NMP-Q			
Absence of nomophobia	16 (0.59%)	2 (0.66%)	0.8
Mild nomophobia	706 (26.09%)	48 (15.68%)	
Moderate nomophobia	1394 (51.52%)	150 (49.01%)	
Severe nomophobia	590 (21.8%)	106 (34.64%)	
AIS			
Healthy	1177 (43.50%)	--	
Insomnia	1529 (56.50%)	--	
GAD-7			
Minimal anxiety	602 (22.25%)	--	
Mild anxiety	1104 (40.80%)	--	
Moderate anxiety	663 (24.50%)	--	
Severe anxiety	337 (12.45%)	--	

Note: Results presented as mean ± standard deviation for continuous data or frequency counts and percentage for categorical data; MPU = mobile phone use; NMP-Q = Nomophobia Questionnaire (NMP-Q); AIS = Athens Insomnia Scale, GAD-7 = General Anxiety Disorder-7. T1 = at baseline; T2 = during social media outage. -- = Not measured. * Difference between groups based on independent samples *t*-test/χ^2^.

**Table 2:** Nomophobia scores at baseline and during the social media outage.

Variable	T1 (N = 2706)	T2 (N = 306)	Mean Difference	*p*	Cohen’s d
(D1) Not being able to communicate	4.05 ± 1.61	4.47 ± 1.62	0.63	<0.001	0.42
(D2) Losing connectedness	3.33 ± 1.68	3.99 ± 1.65	0.83	<0.001	0.53
(D3) Not being able to access information	4.24 ± 1.51	4.83 ± 1.47	0.76	<0.001	0.54
(D4) Giving up convenience	3.91 ± 1.56	4.47 ± 1.46	0.76	<0.001	0.51
NMP-Q Score	77.38 ± 26.38	88.46 ± 26.54	14.81	<0.001	0.57

Note: Paired samples *t*-test; significance *p* < 0.05; T1 = at baseline; T2 = during social media outage; NMP-Q = Nomophobia Questionnaire (NMP-Q), D1–D4 NMP-Q dimensions.

**Table 3:** The association between study variables.

Variable	MPU	NMP-Q (T1)	NMP-Q (T2)	GAD-7	AIS
MPU	—				
NMP-Q (T1)	*r* = 0.11; *p =* 0.001	—			
NMP-Q (T2)	*r* = 0.09; *p =* 0.09	*r* = 0.53; *p =* 0.001	—		
GAD-7	*r* = 0.05; *p =* 0.001	*r* = 0.14; *p =* 0.001	*r* = 0.05; *p =* 0.40	—	
AIS	*r* = 0.09; *p =* 0.001	*r* = 0.09; *p =* 0.001	*r* = 0.02; *p =* 0.70	*r* = 0.25; *p =* 0.001	—

Note: *r* = Pearson product–moment correlation; significance *p* < 0.05; MPU = mobile phone use, h/d; NMP-Q = Nomophobia Questionnaire (NMP-Q); AIS = Athens Insomnia Scale; GAD-7 = General Anxiety Disorder-7; T1 = at baseline; T2 = during social media outage. Sample size at T1 = 2706 and at T2 = 306.

**Table 4:** The association between AIS and GAD-7 scores at baseline (T1) with the change in NMP-Q symptoms during the social media outage (T2–T1).

NMP-Q Symptoms during Social Media Outage (T2–T1)	β	SE	*p*-Value
AIS (T1)	−0.78	0.33	0.02 *
GAD (T1)	−0.38	0.32	0.20

Note: NMP-Q = Nomophobia Questionnaire (NMP-Q); AIS = Athens Insomnia Scale; GAD-7 = General Anxiety Disorder-7. Outcome variable NMP-Q symptoms during the social media outage (T2–T1). β = unstandardized beta, * significant at *p* < 0.05.

**Table 5:** The association between AIS and GAD-7 scores at baseline (T1) with the change in the four dimensions of the NMP-Q symptoms during the social media outage (T2–T1).

NMP-Q Dimension 1: (D1) Not Being Able to Communicate (T2–T1)	β	SE	*p*-Value
AIS (T1)	−0.03	0.02	0.10
GAD (T1)	−0.03	0.02	0.08
NMP-Q Dimension 2: (D2) Losing connectedness			
AIS (T1)	−0.04	0.02	0.08
GAD (T1)	−0.01	0.02	0.70
NMP-Q Dimension 3: (D3) Not being able to access information			
AIS (T1)	−0.04	0.02	0.03 *
GAD (T1)	−0.01	0.02	0.40
NMP-Q Dimension 4: (D4) Giving up convenience			
AIS (T1)	−0.05	0.02	0.006 *
GAD (T1)	−0.02	0.02	0.30

Note: NMP-Q = Nomophobia Questionnaire (NMP-Q); AIS = Athens Insomnia Scale; GAD-7 = General Anxiety Disorder-7. Outcome variable NMP-Q symptoms during the social media outage (T2–T1). β = unstandardized beta, * significant at *p* < 0.05.

## Data Availability

Data are available from the corresponding author based upon request.
